# Identification of unvaccinated children, Madagascar

**DOI:** 10.2471/BLT.25.295299

**Published:** 2026-05-27

**Authors:** Nadine Muller, Apollinaire F Mahafeno, Andrinirina C Rasolofondraibe, Gabrielle T Andriantiantsoa, Andry NS Randrianirisoa, Elgiraud Ramarosaiky, Fanjatiana RLR Rakotoarisoa, Fanilo N Valitera, Zavaniarivo Rampanjato, Miora M Andriamanjay, Elsa N Rajemison, Riana S Ramanantsoa, Julius V Emmrich, Lara M Press, Christian H Ravelomahafaly, Maurin Mananatitra

**Affiliations:** a Department of Infectious Diseases and Critical Care Medicine, Charité – Universitätsmedizin Berlin, Charitéplatz 1, 10117 Berlin, Germany.; b Doctors for Madagascar, Antananarivo, Madagascar.; c mTOMADY, Antananarivo, Madagascar.; d Center for Global Health, Charité – Universitätsmedizin Berlin, Berlin, Germany.

## Abstract

**Problem:**

Madagascar has a high number of unvaccinated (zero-dose) children. In 2023, an estimated 74% received any dose of the diphtheria–tetanus–pertussis (DTP) vaccine. In southern Madagascar, service gaps, poverty, drought and limited access leave many children unreached by routine immunization.

**Approach:**

Within the *Sorohy ny Aretina Manaova Ezaka Vaksiny* initiative, a community-based enumeration and vaccination-status verification was conducted in 16 districts in August 2024–June 2025 under health ministry authorization. A total of 3975 community health workers (CHWs) were recruited and trained to verify the vaccination status of all children younger than 5 years from available documentation in targeted *fokontany* (lowest administrative unit). Digital tools supported data collection, validation and CHW incentive payments.

**Local setting:**

The southern regions of Atsimo Andrefana, Androy and Anosy have about 3.7 million inhabitants, most in remote areas. Health indicators lag behind national averages. Volunteer CHWs lack training, supervision and financial protection, resulting in incomplete population data and weak microplanning.

**Relevant changes:**

Among the *fokontany*, 81% (3538/4347) completed registry updating. Overall, 58% (213 173/368 227) of the children enumerated had no documented DTP dose. District-level prevalence of no-dose children ranged from 25% (7956/32 015) to 86% (14 989/17 445). These data guided zero-dose mapping and targeted outreach. As of March 2026, 42 700 zero-dose children had received DTP vaccination, surpassing the programme target ahead of schedule.

**Lessons learnt:**

Locally led partnerships and CHW engagement, supported by simple digital tools and transparent financial mechanisms, produced actionable data on zero-dose children. Prior agreement on zero-dose case definitions ensures compatibility with national reporting.

## Résumé

**Problème:**

Madagascar compte un nombre élevé d’enfants non vaccinés. En 2023, on estimait que 74% des enfants avaient reçu au moins une dose du vaccin contre la diphtérie, le tétanos et la coqueluche (DTC). Dans le sud de Madagascar, les lacunes dans les services, la pauvreté, la sécheresse et un accès limité empêchent de nombreux enfants de bénéficier de la vaccination systématique.

**Approche:**

Dans le cadre de l’initiative *Sorohy ny Aretina Manaova Ezaka Vaksiny*, un recensement communautaire et une vérification du statut vaccinal ont été menés dans 16 districts entre août 2024 et juin 2025, sous l’égide du ministère malgache de la Santé. En tout, 3 975 agents de santé communautaires (ASC) ont été recrutés et formés pour vérifier le statut vaccinal de tous les enfants de moins de 5 ans à partir des documents disponibles dans les *fokontany* (unités administratives de base) ciblées. Des outils numériques ont facilité la collecte et la validation des données, ainsi que le versement des primes aux ASC.

**Environnement local:**

Les régions méridionales d’Atsimo-Andrefana, d’Androy et d’Anôsy comptent environ 3,7 millions d’habitants, dont la plupart vivent dans des zones reculées. Les indicateurs de santé y sont inférieurs aux moyennes nationales. Les ASC bénévoles manquent de formation, de supervision et de protection financière, ce qui se traduit par des données démographiques incomplètes et une planification locale déficiente.

**Changements significatifs:**

Parmi les *fokontany*, 81% (3 538 sur 4 347) ont achevé la mise à jour des registres. En tout, 58% (213 173 sur 368 227) des enfants recensés n’avaient aucune dose de vaccin DTC documentée. Au niveau des districts, la prévalence des enfants non vaccinés variait de 25% (7 956 sur 32 015) à 86% (14 989 sur 17 445). Ces données ont guidé la cartographie des enfants n’ayant reçu aucune dose de vaccin et les actions de sensibilisation ciblées. En mars 2026, 42 700 enfants n’ayant reçu aucune dose avaient reçu le vaccin DTC, dépassant ainsi l’objectif du programme avant la date prévue.

**Leçons tirées:**

Les partenariats menés au niveau local et l’engagement des ASC, soutenus par des outils numériques simples et des mécanismes financiers transparents, ont permis d’obtenir des données exploitables sur les enfants n’ayant reçu aucune dose de vaccin. Un accord préalable sur les définitions des cas d’enfants n’ayant reçu aucune dose garantit la compatibilité avec les rapports nationaux.

## Resumen

**Situación:**

Madagascar tiene un número elevado de niños no vacunados (dosis cero). En 2023, se estimó que el 74% recibió alguna dosis de la vacuna contra la difteria, el tétanos y la tos ferina (DTP). En el sur de Madagascar, las deficiencias en los servicios, la pobreza, la sequía y el acceso limitado hacen que muchos niños queden fuera de la inmunización sistemática.

**Enfoque:**

En el marco de la iniciativa *Sorohy ny Aretina Manaova Ezaka Vaksiny*, se llevó a cabo un registro comunitario y una verificación del estado de vacunación en 16 distritos entre agosto de 2024 y junio de 2025, con autorización del ministerio de salud. Se reclutó y formó a un total de 3975 trabajadores comunitarios de salud (TCS) para verificar el estado de vacunación de todos los niños menores de 5 años a partir de la documentación disponible en los *fokontany* (unidad administrativa más pequeña) seleccionados. Herramientas digitales apoyaron la recopilación y la validación de datos, así como el pago de incentivos a los TCS.

**Marco regional:**

Las regiones meridionales de Atsimo Andrefana, Androy y Anosy tienen aproximadamente 3,7 millones de habitantes, la mayoría en zonas remotas. Los indicadores sanitarios se sitúan por debajo de los promedios nacionales. Los TCS voluntarios carecen de formación, supervisión y protección financiera, lo que da lugar a datos poblacionales incompletos y a una microplanificación deficiente.

**Cambios importantes:**

Entre los *fokontany*, el 81% (3538/4347) completó la actualización de registros. En conjunto, el 58% (213 173/368 227) de los niños registrados no tenía ninguna dosis de DTP documentada. La prevalencia de niños con dosis cero a nivel distrital osciló entre el 25% (7956/32 015) y el 86% (14 989/17 445). Estos datos orientaron el mapeo de niños con dosis cero y las actividades de alcance dirigidas. En marzo de 2026, 42 700 niños con dosis cero habían recibido la vacuna DTP, superando antes de lo previsto el objetivo del programa.

**Lecciones aprendidas:**

Las alianzas lideradas a nivel local y la participación de los TCS, apoyadas por herramientas digitales sencillas y mecanismos financieros transparentes, generaron datos útiles para la acción sobre los niños con dosis cero. El acuerdo previo sobre las definiciones de casos de dosis cero asegura la compatibilidad con los sistemas nacionales de notificación.

## ملخص

**المشكلة:**

تعاني مدغشقر من نسبة مرتفعة للأطفال الذين لم يحصلوا على اللقاح (لم يتلقوا أية جرعة). في عام 2023، تلقى ما يقدر بـ 74% منهم جرعة واحدة من لقاحات الدفتيريا والتيتانوس والسعال الديكي (DTP). في جنوب مدغشقر، يتعرض العديد من الأطفال للحرمان من التحصين الروتيني بسبب نقص الخدمات والفقر والجفاف وفرص الوصول المحدودة.

**الأسلوب:**

في إطار مبادرة *Sorohy ny Aretina Manaova Ezaka Vaksiny *، تم إجراء إحصاء مجتمعي، وتحقق من حالة التلقيح في 16 مقاطعة خلال الفترة من أغسطس/آب 2024 إلى يونيو/حزيران 2025 بموجب تفويض من وزارة الصحة. تم توظيف وتدريب 3975 من العاملين في مجال الخدمات الصحية المجتمعية (CHW) للتحقق من حالة اللقاح لجميع الأطفال دون سن الخامسة من خلال الوثائق المتاحة في وحدات *fokontany * المستهدفة (أصغر وحدة إدارية). تم استخدام الأدوات الرقمية لدعم جمع البيانات، وتحقيقها، ودفع حوافز العاملين في مجال الخدمات الصحية المجتمعية (CHW).

**المواقع المحلية:**

يبلغ عدد سكان المناطق الجنوبية من أتسيمو أندريفانا، وأندروي، وأنوسي حوالي 3.7 مليون نسمة، معظمهم في مناطق نائية. وتتأخر المؤشرات الصحية خلف المتوسطات الوطنية. ويفتقر العاملون المتطوعون في مجال الخدمات الصحية المجتمعية (CHW) إلى التدريب والإشراف والحماية المالية، مما يؤدي إلى نقص في بيانات السكان وضعف التخطيط الجزئي.

**التغيّرات ذات الصلة:**

من بين وحدات *fokontany *، أكمل 81% (3538/4347) تحديث سجلاتهم. وبشكل عام، لم يحصل 58% (213173/368227) من الأطفال الذين تم إحصاؤهم أية جرعة موثقة من لقاحات الدفتيريا والتيتانوس والسعال الديكي (DTP). وتراوحت نسبة انتشار الأطفال الذين لم يتلقوا أي جرعة على مستوى المقاطعات من 25% (7956/32015) إلى 86% (14989/17445). وقد قامت هذه البيانات بتوجيه عملية تحديد المناطق التي لم تحصل على أية جرعة، والحملات المستهدفة. بدءًا من مارس/آذار 2026، فإن الـ 42700 طفلاً الذين لم يحصلوا على أي لقاح، قد تلقوا لقاح الدفتيريا والتيتانوس والسعال الديكي (DTP)، متجاوزين بذلك هدف البرنامج قبل الموعد المحدد.

**الدروس المستفادة:**

إن الشراكات المحلية، ومشاركة العاملين في مجال الخدمات الصحية المجتمعية (CHW)، مع دعم من أدوات رقمية بسيطة وآليات مالية شفافة، قد أسفرت عن بيانات قابلة للاستخدام والتخطيط بشأن الأطفال الذين لم íÍÕáæÇ Úáì ÃíÉ ÌÑÚÉ. Åä ÇáÇÊÝÇÞ ÇáãÓÈÞ ÈÎÕæÕ ÊÚÑíÝ ÇáÍÇáÇÊ ÇáÊí áã ÊÍÕá Úáì ÃíÉ ÌÑÚÇÊ¡ íÖãä ÇáÊæÇÝÞ ãÚ ÅÚÏÇÏ ÇáÊÞÇÑíÑ ÇáæØäíÉ.

## 摘要

**问题:**

在马达加斯加，未接种疫苗儿童（零剂量儿童）的占比很高。2023 年，无任何白喉-破伤风-百日咳 (DTP) 疫苗接种记录的儿童约占 74%。在马达加斯加南部地区，因服务缺口、贫困、干旱和可及性受限等原因而导致许多儿童无法定期接种疫苗。

**方法:**

根据“接种疫苗，远离疾病 (*Sorohy ny Aretina Manaova Ezaka Vaksiny*) 倡议，2024 年 8 月至 2025 年 6 月期间，经卫生部授权，有 16 个地区开展了基于社区的查点和疫苗接种状态核查活动。被招募而来的总计 3975 名社区卫生工作者 (CHW) 接受培训后，在目标社区 (*fokontany*)（最低行政单位）中根据现有接种记录，核查所有 5 岁以下儿童的疫苗接种状态。数字工具支持数据收集、验证和 CHW 激励性报酬支付。

**当地状况:**

马达加斯加南部的阿齐莫-安德列发娜区、安德罗伊区和阿诺西区共有约 370 万居民，且大部分生活在偏远地区。卫生指标低于国家平均水平。志愿者性质的 CHW 未能接受培训监管和获得财务保障，从而导致人口数据不完整且微观计划薄弱。

**相关变化:**

在社区 (*fokontany*) 中，有 81% (3538/4347) 的儿童完成了登记更新。总体来说，在接受查点的儿童中，有 58% (213173/368227) 无 DTP 疫苗接种记录。各地区零剂量儿童的比例分别为 25% (7956/32015) 至 86% (14989/17445) 不等。这些数据能够为确定零剂量儿童分布和有针对性地推行外展服务提供指导。截至 2026 年 3 月，42700 名零剂量儿童完成了 DTP 疫苗接种，这表示提前超目标完成了计划任务。

**经验教训:**

通过当地主导开展合作、招募 CHW 有效参与、利用简单数字工具以及实施透明财务机制，生成了有关零剂量儿童的可操作数据。事先约定零剂量儿童的定义确保最终结果与国家报告体系相兼容。

## Резюме

**Проблема:**

На Мадагаскаре многие дети не были охвачены вакцинацией (дети с нулевой дозой). В 2023 году около 74% детей получили хотя бы одну дозу вакцины от коклюша, столбняка и дифтерии (КДС). На юге Мадагаскара многие дети остаются не охваченными плановой иммунизацией из-за пробелов в предоставлении услуг, бедности, засухи и затрудненного доступа.

**Подход:**

В период с августа 2024 года по июнь 2025 года в рамках инициативы *Sorohy ny Aretina Manaova Ezaka Vaksiny* и с разрешения Министерства здравоохранения на базе отдельных общин был проведен поименный учет и определение статуса вакцинации населения в 16 районах. Было нанято и проинструктировано в общей сложности 3975 общинных медицинских работников, которые проверяли статус вакцинирования всех детей в возрасте до 5 лет в целевых *фоконтанах* (низших административных единицах). Цифровые инструменты обеспечивали сбор и проверку данных, а также выплату бонусов общинным медицинским работникам.

**Местные условия:**

В южных регионах Андруй, Ануси и Ациму-Андрефана проживают около 3,7 миллиона жителей, по большей части в труднодоступной местности. Показатели здравоохранения здесь отстают от средних по стране. Общинным медицинским работникам, работающим на добровольных началах, недостает обучения, надзора и финансовой защиты, что приводит к неполноте данных о населении и слабому микропланированию.

**Осуществленные перемены:**

В 81% *фоконтанов* (3538 из 4347) были обновлены реестры. В целом 58% (213 173 из 368 227) выявленных детей не имели документального подтверждения получения дозы КДС. По отдельным районам количество невакцинированных детей колебалось от 25% (7956 из 32 015) до 86% (14 989 из 17 445). Эти данные использовались для картирования лиц с нулевой дозой вакцинации и адресной работы с ними. По состоянию на март 2026 года 42 700 невакцинированных детей получили вакцину КДС, тем самым цель программы была перевыполнена досрочно.

**Выводы:**

Партнерства под местным руководством и участие общинных медицинских работников, поддерживаемые простыми цифровыми инструментами и прозрачными финансовыми механизмами, позволили получить практические данные о детях с нулевой дозой вакцинации. Предварительное согласование определений случаев нулевой дозы обеспечивает совместимость с национальной системой отчетности.

## Introduction

Routine childhood immunization is among the most effective and affordable public health measures for reducing child deaths.^1^ Children who have not received even one dose of a diphtheria–tetanus–pertussis vaccine (DTP1) are considered zero-dose. This categorization is a practical measure to indicate exclusion from basic health services. By definition, the percentage of zero-dose children equals 100% minus DTP1 coverage.^2^ In 2023, Madagascar’s estimated DTP1 coverage was at 74%.^3^

From 2019 to 2022, Madagascar was among the 10 African countries with the largest cumulative number of zero-dose children.^4^ The southern regions of the country are disproportionately affected by poverty, constrained transport access, recurrent drought and food insecurity.^5^^,^^6^ Weak health infrastructure and low community demand for vaccination contributed to recurrent outbreaks of vaccine-preventable disease, including a 2018–2019 measles epidemic and circulating vaccine-derived poliovirus between 2020 and 2023.^7^^,^^8^ A key operational gap is the lack of disaggregated, village-level data on where zero-dose children live to guide targeted outreach.

In 2024, Doctors for Madagascar, a national nongovernmental organization, with the Ministry of Health’s Expanded Programme on Immunization (EPI) and an academic institution, launched *Sorohy ny Aretina Manaova Ezaka Vaksiny* (SOAMEVA) in Malagasy, meaning: prevent diseases, make an effort for vaccination. This initiative aims to reach 40 000 zero-dose children in 16 districts in three southern regions by mid-2026. We describe the initiative’s community-based listing and vaccination status verification designed to locate zero-dose children at household and village levels, thereby enabling targeted outreach and guiding vaccination efforts.

## Local settings

Southern Madagascar (regions of Androy, Anosy and Atsimo-Andrefana) has about 3.7 million inhabitants, many living in remote rural areas with limited access to public services.^9^ Primary health care is delivered through *centres de santé de base* and is supported by community health workers (CHWs). CHWs are volunteer health workers periodically trained by district health authorities. Frequent gaps in training, supervision and financial protection contribute to incomplete paper-based community registers of children and vaccination status, and to unreliable data, complicating vaccination planning and outreach.^10^ SOAMEVA’s 2024 baseline assessment, including stakeholder interviews, a rapid assessment of 375 primary health facilities and a cross-sector workshop, confirmed that local immunization needs were underestimated and informed the design of the community-level enumeration.

The SOAMEVA programme has been scientifically evaluated by assessing the reach, effectiveness, adoption, implementation and sustainability of all programme components. This evaluation is ongoing and described in detail elsewhere.^11^

## Approach

The enumeration was designed as a programme tool, not a statistical survey, to generate data on where zero-dose children live to guide outreach and microplanning. Conducted between August 2024 and June 2025 in 16 districts, the activity was coordinated by Doctors for Madagascar with regional and district health authorities. CHWs were recruited to undertake the enumeration using the health ministry’s standard community registry. Four steps were followed: registration, training and mobilization; household visits; verification and cross-checking; and digital validation and payment.

In step 1, each primary health facility selected one CHW per served *fokontany* (lowest administrative unit) who was registered to Tsaracheck, a digital payment and verification tool using subscriber identity module (SIM) card-linked unique identifiers. These CHWs received a half-day training on household visits, vaccination status verification and registry updating, with training content aligned with national EPI protocols.

In step 2, CHWs visited all households over 5 days, listing every child younger than 5 years and recording vaccination status from available documentation. If no household member was present, CHWs asked neighbours about the family’s whereabouts and left a message requesting a return visit. CHWs documented vaccination status for all pathogens in the national schedule. We report DTP as the main zero-dose indicator. Doctors for Madagascar undertook supervision by spot checks.

In step 3, Doctors for Madagascar staff, together with facility staff and CHWs where available, verified and cross-checked completed registries against facility vaccination records from the preceding 5 years. A child was classified as zero-dose only if no documentary evidence of DTP vaccination was found from all available sources. Doctors for Madagascar staff and the facility head co-signed a cumulative report summarizing, for each *fokontany*, the number of children and the number classified as zero-dose. Non-availability of facility heads necessitated repeated visits sometimes.

In step 4, Doctors for Madagascar staff scanned and uploaded validated registries to Tsaracheck. Aggregated data per *fokontany* were entered into CommCare® (Cambridge, United States of America) for analysis and mapping, conditional on the co-signed verification report. CHWs received 50 000 Malagasy ariary (Ar) – about 11 United States dollars (US$) – on successful completion, equivalent to the government’s monthly cap for CHW incentives. Payments were processed and transferred via Tsaracheck after validation by the nongovernmental organization’s claims and finance team. The mobile-money transfer fee per payment (Ar 1300; about US$ 0.30) was covered by the programme and not deducted from the CHW incentive.

The enumeration was launched sequentially by district cohort: nine districts between August 2024 and April 2025 and seven districts between February and June 2025; no district appeared in both phases. A dedicated field team managed each district, supported by colleagues from districts that had already completed or not yet started enumeration. Vaccination status was recorded on the enumeration date.

The activity had a budget of 380 000 euros (€) – US$ 418 000 – including all human resources and field expenses. Digital tools were additional costs, including a paid CommCare® Pro plan (monthly subscription €400; US$ 430) and €30 000 (US$ 33 000) for Tsaracheck set-up and maintenance. The enumeration was conducted as a routine public health activity formally authorized by the health ministry. Individual-level data are stored on a protected server accessible only to authorized staff; no personal identifiers are reported here.

## Relevant changes

In total, 4407 people were mobilized for the enumeration: 82 nongovernmental organization staff, 22 government health officials, 328 facility-based health workers and 3975 CHWs.

The activity covered 97% (328/339) of planned primary care facilities. Of targeted *fokontany*, 81% (3538/4347) completed registry updating, confirmed by the *fokontany* head. Data reported hereafter reflect only these *fokontany*. Data from 74% (3217/4347) of all targeted *fokontany* were digitized. Verified mobile payments were made to 3514 CHWs totalling Ar 180 million (about US$ 39 500) through Tsaracheck.

Overall, 58% (213 173/368 227) of the children enumerated had no documented DTP dose (operational definition of zero-dose). District-level prevalence ranged from 25% (7956/32 015) in Fort-Dauphin to 86% (14 989/17 445) in Beloha, with the largest numbers in Ampanihy (45 676 children) and Betioky (30 682 children; Table 1). These data guided local zero-dose mapping and targeted outreach.

**Table 1:** Percentage of children 0–59 months with zero doses of diphtheria–tetanus–pertussis vaccine, by region and district, Southern Madagascar, 2024–2025

Region and district	No. of children identified	No. of zero-dose children, (%)
**Androy**		
Ambovombe	18 681	12 075 (65)
Bekily	35 407	22 493 (64)
Beloha	17 445	14 989 (86)
Tsihombe	18 804	9 171 (49)
Subtotal	90 337	58 728 (65)
**Anosy**		
Amboasary	39 231	18 602 (47)
Betroka	17 484	13 956 (80)
Fort Dauphin	32 015	7 956 (25)
Subtotal	88 730	40 514 (46)
**Atsimo Andrefana **		
Ampanihy	62 584	45 676 (73)
Ankazoabo	7 077	2 398 (34)
Benenitra	3 842	2 379 (62)
Beroroha	4 386	3 014 (69)
Betioky	44 611	30 682 (69)
Morombe	11 303	8 243 (73)
Sakaraha	10 499	8 713 (83)
Toliara I	5 163	2 239 (43)
Toliara II	39 695	10 587 (27)
Subtotal	189 160	113 931 (60)
**Total**	**368 227**	**213 173** (**58**)

Note: Data included are from *fokontany* (lowest administrative unit) with complete updated registries as confirmed by the *fokontany *head (81%; 3538/4347 of targeted *fokontany*). The figures therefore represent operational proportions among enumerated children, not population-level estimates.

Enumeration revealed a substantially higher zero-dose burden than anticipated by previous estimates. The resulting evidence guided targeted sensitization and mobile vaccination efforts across 16 districts. As of March 2026, 42 700 zero-dose children in all 16 districts had been reached with DTP1 vaccination, surpassing the programme target ahead of schedule. Additionally, more than 90 000 children younger than 5 years had received at least one vaccine dose. While SOAMEVA’s vaccination activities are ongoing until July 2026 and georeferenced data have been shared with local health authorities to guide routine services and campaigns, an extension to reach remaining zero-dose children is being pursued, pending funding approval.

## Lessons learnt

The SOAMEVA initiative demonstrated how jointly defined district priorities, CHW engagement and digital systems can identify zero-dose children and guide programme activities at scale (Box 1). The integrated paper-based and digital data collection process provided a geographically disaggregated view of where zero-dose children were concentrated. In addition, the availability of digital scanned community registries created a unique database of individual immunization records, enabling an ongoing secondary analysis of factors associated with vaccination coverage.

Box 1 **Summary of main lessons learnt**

- Enabling community health workers and ensuring fair, direct payment via mobile money were decisive for success.
- Combining paper registries with simple digital tools provided precise evidence on where zero-dose children were located.
- Explicit prior agreement on zero-dose case definitions with national authorities is essential to ensure compatibility of results with national reporting systems.

CHW dedication was decisive as their community ties and commitment to child health ensured acceptance and completeness of the enumeration. Transparent incentive payments to personal accounts, initially viewed with hesitation, proved effective and built confidence. This approach offers a practical model for equitable compensation in large, community-based interventions. Joint needs assessment and alignment with district workplans secured ownership and ensured consistent validation by public health authorities.

Two main challenges emerged. First, when new data revealed substantially higher zero-dose figures than previous estimates, some health facility leads hesitated to report them. District health authorities conducted site visits to reframe higher figures as evidence of improved data quality (rather than poor past performance). We learnt that changing established views about data requires sustained engagement. Future activities should strengthen communication to help health workers recognize discrepancies as improved accuracy. Second, differences in zero-dose definitions created challenges. SOAMEVA classified a child as zero-dose only if no DTP vaccination evidence was found in household, community and facility records. This definition is more rigorous than approaches using only vaccination cards but can produce higher estimates of zero-dose children than recall-inclusive methods. Although the documentation-based zero-dose definition was initially approved by the EPI, it later shifted its definition to include caregiver recall. However, because Madagascar’s community registry does not include recall, this information was not collected during enumeration. Consequently, the collected data and results were not integrated into the national DHIS2 system. Nevertheless, the georeferenced *fokontany*-level enumeration results directly guided SOAMEVA’s and other local actors’ targeted vaccination and community sensitization activities. Future activities should agree on case definitions at the start and, ideally, collect both documentation and recall data.

Several elements of our approach are transferable: documentation-based zero-dose identification; mobile-money incentives; community registry updating for pathogen-level targeting; and joint planning between nongovernmental organizations and government. The logistical challenges of geographic dispersion and community dynamics are in part specific to Southern Madagascar.

Three limitations should be noted. First, children without any vaccination documentation were classified as zero-dose regardless of recall; true unvaccinated prevalence may be lower. Second, enumeration completeness was not independently verified, and the denominator was restricted to enumerated children in *fokontany* that completed registry updating; results therefore represent operational proportions, not population-level estimates. *Fokontany* where registry updating was not completed were likely more remote and harder to reach. The data may therefore underestimate the true zero-dose burden. Third, reporting bias cannot be excluded. However, as our zero-dose figures substantially exceed previous estimates, underreporting driven by institutional desirability at the facility level is a more plausible bias than overreporting.
